# Revealing the Power of the Natural Red Pigment Lycopene 

**DOI:** 10.3390/molecules15020959

**Published:** 2010-02-23

**Authors:** Kin-Weng Kong, Hock-Eng Khoo, K. Nagendra Prasad, Amin Ismail, Chin-Ping Tan, Nor Fadilah Rajab

**Affiliations:** 1 Department of Nutrition and Dietetics, Faculty of Medicine and Health Sciences, Universiti Putra Malaysia, 43400 UPM Serdang, Selangor, Malaysia; E-Mails: kchai21@yahoo.com (K.-W.K.); hockeng_khoo@yahoo.com (H.-E.K.); knag76@gmail.com (K.N.P.); 2 Laboratory of Analysis and Authentication, Halal Products Research Institute, Universiti Putra Malaysia, 43400 Serdang, Selangor, Malaysia; 3 Department of Food Technology, Faculty of Food Science and Technology, Universiti Putra Malaysia, 43400 Serdang, Selangor, Malaysia; E-Mail: tancp@putra.upm.edu.my (C.-P.T.); 4 Department of Biomedical Science, Faculty of Allied Health Sciences, Universiti Kebangsaan Malaysia, Jalan Raja Muda Abdul Aziz, 50300 Kuala Lumpur, Malaysia; E-Mail: nfadilah@medic.ukm.my (N.F.R.)

**Keywords:** lycopene, properties, antioxidant, diseases, by-product

## Abstract

By-products derived from food processing are attractive source for their valuable bioactive components and color pigments. These by-products are useful for development as functional foods, nutraceuticals, food ingredients, additives, and also as cosmetic products. Lycopene is a bioactive red colored pigment naturally occurring in plants. Industrial by-products obtained from the plants are the good sources of lycopene. Interest in lycopene is increasing due to increasing evidence proving its preventive properties toward numerous diseases. *In vitro, in vivo* and *ex vivo* studies have demonstrated that lycopene-rich foods are inversely associated to diseases such as cancers, cardiovascular diseases, diabetes, and others. This paper also reviews the properties, absorption, transportation, and distribution of lycopene and its by-products in human body. The mechanism of action and interaction of lycopene with other bioactive compounds are also discussed, because these are the crucial features for beneficial role of lycopene. However, information on the effect of food processing on lycopene stability and availability was discussed for better understanding of its characteristics.

## 1. Introduction

Natural colored pigments from plant products have drawn great attention worldwide. These pigments display various colors and are made up of different phytochemicals commonly ound in the food matrix such as orange (β-carotene), yellowish-green (lutein), green (chlorophyll), and blue-purple (anthocyanin) [1]. Lycopene is the red colored pigment abundantly found in red colored fruits and vegetables such as tomato, papaya, pink grapefruit, pink guava and watermelon. This red colored pigment was first discovered in the tomato by Millardet in 1876 [2]. It was later named lycopene by Schunck [2]. 

Lycopene is a carotenoid hydrocarbon (also called carotene). The extended conjugated double bond system of these compounds is an important feature in the carotenoids responsible for their attractive colors because it forms the light absorbing chromophore [3]. The existence of visible color in these compounds requires at least seven conjugated double bonds. The greater the number of conjugated double bonds, the higher a wavelength value for maximum absorption [4] is observed.

Lycopene is one of the popular pigments highly accepted by food industry as a food additive and also for its health benefits [5,6]. As a red colorant and antioxidant agent, the demand for lycopene is still increasing. According to [5], total world consumption of lycopene was tripled to 15,000 tonnes in 2004 compared to 5000 tonnes in 1995. Thus, alternative sources for the production of natural lycopene are warranted. Previously, *in vitro* and *in vivo* studies exhibited that lycopene has a beneficial role in chronic diseases such as cardiovascular disease, atherosclerosis, cancer and neurodegenerative disorders. However, some studies reported contrasting outcomes. This review offers an overview of the properties of ycopene with recent evidence on its contributions in human health and also provides broad information on lycopene in food processing by-products. 

The importance of natural food additives is given more attention due to an extensive use of the natural ingredients rather than synthetic compounds in food, cosmetics and pharmaceuticals. Meanwhile, the prices of raw materials are increasing and their availability is decreasing. Food processing by-products from orange [7], mango [8,9,10], guava [8,11,12,13,14], pomegranate [15,16], and also vegetables including tomato [17,18,19,20], and carrot [21] are potential sources of functional foods, and at the same time these by-products are natural sources for lycopene and may have preventive effects against numerous diseases.

## 2. Lycopene in Food and Its Properties

Lycopene is an unsaturated acyclic carotenoid with 11 linear conjugated and two non-conjugated double bonds. It is not the precursor for vitamin A, since it lacks the terminal β-ionic ring found in the basic structure of vitamin A. The red color of certain fruits and vegetables such as tomato, pink grapefruit, red grapes, watermelon and red guava is due to the presence of lycopene. Lycopene is reported as the most efficient singlet oxygen quencher in carotenoids group, whose quenching ability is mainly dependent on the number of conjugated double bonds, and to a lesser influenced by either the presence of cyclic or acyclic end groups [22]. In addition, its chain structure with an extensive conjugated polyene system is important for its biological properties such as susceptibility to oxidative degradation [23]. 

**Figure 1:**
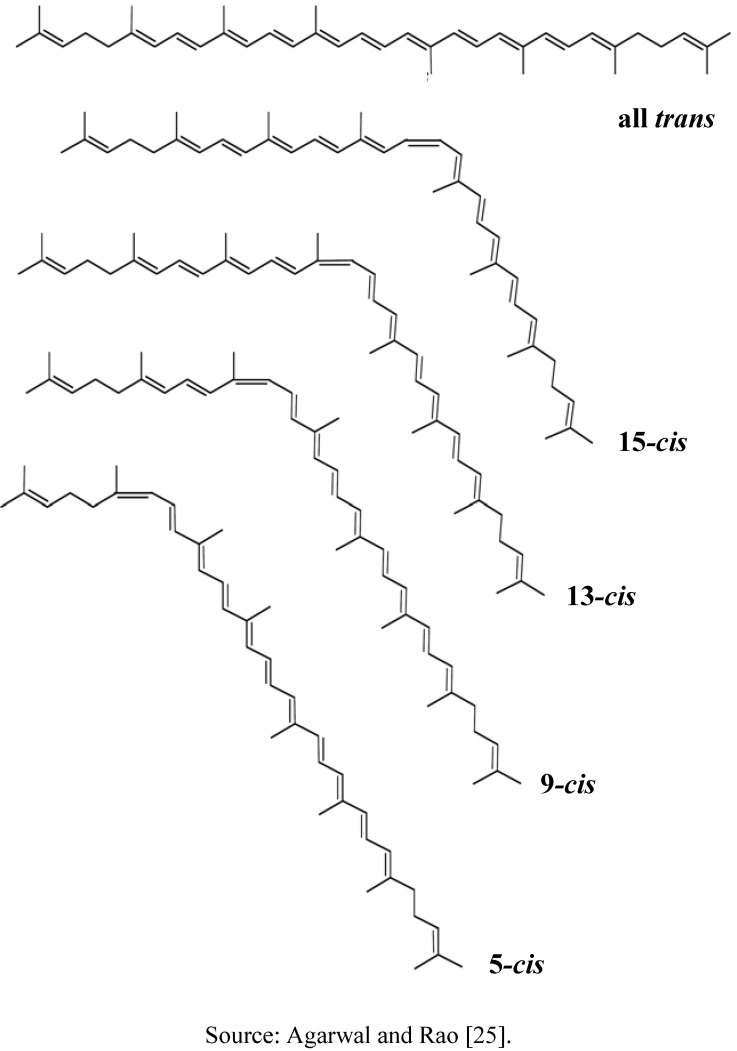
Molecular structures of lycopene isomers.

Lycopene occurs naturally as all *trans* form and its chain containing seven double bonds that can be isomerized to mono-*cis* or poly-*cis* due to the exposure to high temperatures, light, oxygen, acids, catalyst and metal ions [23]. Lycopene is a lipophilic compound with hydrophobic characteristics due to its acyclic structure and 11 linear conjugated double bonds that make it more soluble in organic solvents such as chloroform, hexane, benzene, methylene chloride, acetone and petroleum ether [24]. Physical properties and molecular structure of lycopene are shown in Table 1 and Figure 1, respectively. 

**Table 1:** Physical properties of lycopene.

Molecular formula	C_40_H_56_
Molecular weight	536.85 Da
Melting point	172–175 ºC
Crystal form	Long red needles separate from a mixture of carbon disulfide and ethanol
Powder form	Dark reddish-brown
Solubility	Soluble in chloroform, hexane, benzene, carbon disulfide, acetone, petroleum ether and oil;
	Insoluble in water, ethanol and methanol
Stability	Sensitive to light, oxygen, high temperature, acids, catalyst and metal ions

Source: Shi *et al.* [23].

## 3. Lycopene Absorption, Transportation and Distribution in Human

### 3.1. Absorption

As a fat soluble compound, lycopene has a similar absorption as dietary fat. In the stomach and duodenum, lycopene will separate from the food matrix and subsequently dissolve in the lipid phase [26]. Prior to absorption, the lipid phase will form droplets, resulting from the reaction with bile salts and pancreatic lipases. Then, it enters the duodenum and appears as the multi-lamellar lipid vesicles [27]. Finally, the lipid vesicles will absorb into small intestine via passive or diffusion process [26]. Additionally, there are *in vitro* studies suggested that the intestinal absorption of lycopene was aided by the participation of a specific epithelial transporter [28,29]. 

The absorption of lycopene was reported to be lower compared to other carotenoids based on an *in vitro* study using the Caco-2 cell line [29]. However, there are many factors that might affect the absorption of lycopene. The degree of lycopene release from the food matrix into the digestive tract will be lowered when the indigestible fraction increases [30]. High fibers diets will reduce the uptake of lycopene and decrease lycopene adsorption whereby lycopene supplemented together with different dietary fibers has resulted in the reduction of plasma lycopene for more than 40% [31]. The bioaccessibility of lycopene in the intestine has also discussed by Goñi *et al.* [30] who showed that the release of lycopene was higher in the large intestine (57%) than the small intestine (40%), but the potential for lycopene to be absorbed in the large intestine is negligible. Furthermore, an *in vitro* study using Caco-2 cells showed that the uptake of *cis* lycopene was significantly greater than for all *trans* isomer [32]. Thus, *cis* isomers have higher bioavailability than all *trans*-isomers. 

The nature of the human body is believed to cause the isomerization of lycopene along the digestive tract. A study reported that 60% of *cis* lycopene isomers occurred in human plasma, even though the early consumption of the lycopene rich food that mostly consisted of all *trans* lycopene (>90%) [33]. An *in vivo* study also explained that the acidic condition in gastric milieu will enhance isomerization of the all *trans* lycopene to *cis* isomers [34]. This mechanism will further improve the absorption of lycopene once the lycopene reaches the small intestine. 

Food processing is one of the factors which can affect the bioavailability of lycopene, and thus increase absorption. The heating of tomato sauce purposely to induce isomerization of all *trans* lycopene to *cis* isomers could increase the bioavailability of lycopene [35]. Furthermore, *in vitro* study showed sun dried tomatoes give the highest bioavailabiliy of lycopene as compared to fresh and canned tomatoes [36]. Besides, ingestion of lycopene together with oil would also help in increasing its bioavailability [37]. A human study shown that a combination of salad dressing and canola oil increased lycopene content in plasma chylomicrons as compared to fat free salad dressing [38]. This is in agreement with the results of Fielding *et al.* [39] showing that tomatoes cooked with olive oil greatly increase the lycopene level in human plasma as compared to the tomatoes cooked without olive oil. Moreover, bioavailability of lycopene was found to be impaired in elderly people [40].

### 3.2. Transportation

After the uptake by intestinal mucosa, lycopene will be parceled into triacylglycerol-rich chylomicrons and will be secreted into lymph transport system, and lastly transferred to the liver [24]. Lycopene is prone to accumulate in the lipophilic compartments of membrane or lipoprotein [41]. It is transported by plasma lipoproteins and the distribution depends on its chemical structure. As a hydrophobic compound, lycopene is found at the lipophilic part of lipoproteins which is the core of the lipoprotein [27], while other polar carotenoids can be found at the surface of lipoproteins. Therefore, lycopene is mostly transported by low density lipoproteins, while other oxygenated carotenoids are transported by both low density and high density lipoprotein [27]. In addition, *cis* isomers of lycopene were reported to have higher ability to be incorporated in lipoprotein and other protein compared to all *trans* isomer due to the shorter chain length [42]. 

**Table 2:** Plasma lycopene levels in people from different countries.

References	Country	Plasma lycopene levels (µmol/L)	
		Male	Female
[49]	UK	-	0.32 ± 0.12
[50]	USA	0.82 ± 0.38	0.76 ± 0.32
[51]	France	0.66 (0.18-1.47)	0.66 (0.31-2.06)
	Republic of Ireland	0.73 (0.09-2.12)	0.57 (0.09-0.65)
	The Netherland	0.54 (0.08-1.72)	0.53 (0.04-1.98)
	Spain	0.53 (0.21-1.16)	0.51 (0.07-1.72)
	Ireland	0.30 ± 0.13	0.25 ± 0.11
[52]	Italy (Varese/Turin)	1.03 ± 0.43	0.90 ± 0.37
	Italy (Florence)	1.01 ± 0.37	0.90 ± 0.36
	Italy (Ragusa/Naples)	1.29 ± 0.46	1.32 ± 0.46
	Greece (Athens)	0.90 ± 0.38	0.87 ± 0.47
	Spain (Granada)	0.69 ± 0.40	0.69 ± 0.33
	Spain (Murcia)	0.66 ± 0.30	0.74 ± 0.35
	Northern Spain	0.53 ± 0.31	0.43 ± 0.29
	UK (vegetarians)	0.98 ± 0.45	0.89 ± 0.44
	UK (Cambridge)	0.72 ± 0.30	0.77 ± 0.38
	Germany (Potsdam)	0.60 ± 0.30	0.69 ± 0.33
	Germany (Heidelberg)	0.62 ± 0.31	0.54 ± 0.25
	The Netherlands	0.54 ± 0.33	0.47 ± 0.26
	Denmark	0.58 ± 0.34	0.53 ± 0.29
	Sweden (Malmö)	0.46 ± 0.24	0.52 ± 0.27
	Sweden (Umeå)	0.56 ±0.37	0.44 ± 0.25
[53]	Japan	0.11 (0.04-0.33)	0.20 (0.08-0.52)
[54]	Thailand	0.46 ± 0.33	0.74 ± 0.38

### 3.3. Distribution

The distribution of lycopene in human organs and plasma has been reported by Erdman [43], where higher concentrations of lycopene are found in the liver, adrenal and reproductive tissues (ten times higher than other tissues). The concentrations were within the range of 0.2–21.4 nmol/g tissue [44]. Goralczk and Siler [44] reported that lycopene concentration was the highest in human testes, followed by adrenal gland > liver > prostate > breast > pancreas > skin > colon > ovary > lung > stomach > kidney > fat tissue > cervix. A review by Rao and Argawal [6] quoted that lycopene concentrations in human tissues are around 0.15–21.36 nmol/g tissue, but not detectable in brainstem tissue. On the other hand, a study on rats carried out by Zaripheh *et al.* [45] showed that lycopene was highly distributed in the liver. Besides, high lycopene content was found in adipose tissue, the spleen and adrenal tissue. The excretion of lycopene through feces and urine was also reported [45]. 

In human, total serum carotenoids is about 1–2 µM, with lycopene being one of the major carotenoids present in human serum [46]. The level of plasma lycopene can vary among the people from different countries (Table 2). Porrini *et al.* [47] suggested the eating behavior of different individuals makes the lycopene level vary among people. Recently, a study reported that plasma lycopene level could be diverged among married, non-married and divorced subjects [48].

**Figure 2:**
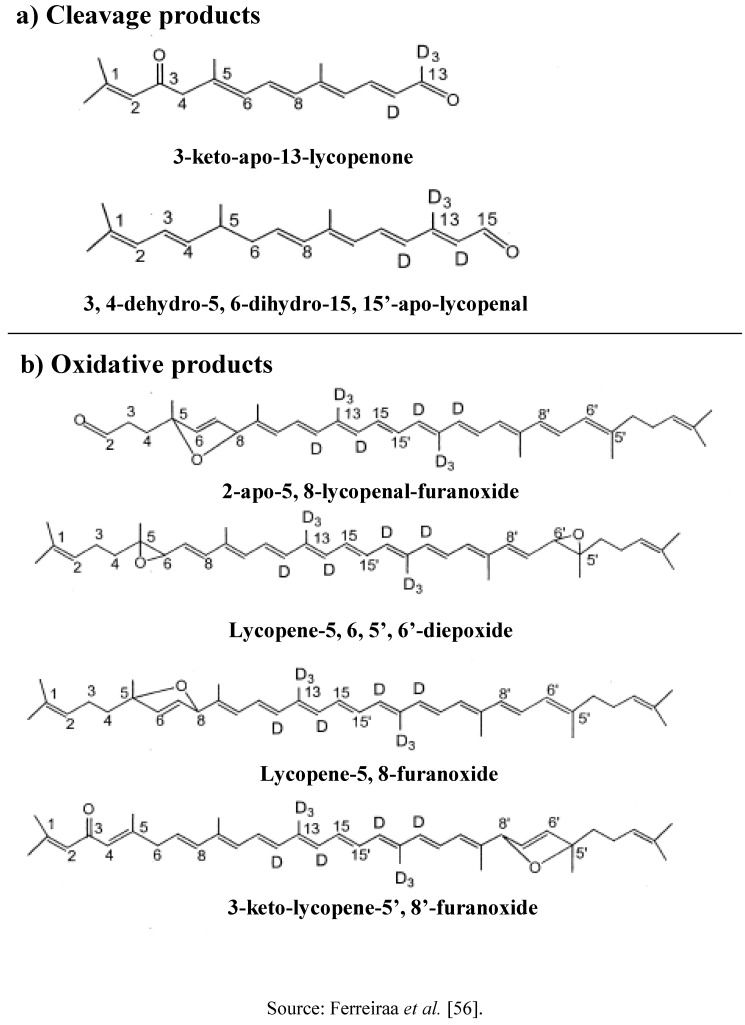
Proposed structures of the metabolites detected.

The lycopene metabolite products were recently studied by Lindshield *et al.* [55] and lycopene metabolites were formed by reacting with carotenoid monooxygenase (CMO) II. Study using post-mitochondrial fraction of rat mucosa with soy lipoxigenase reviewed that cleavage products and oxidation products will be formed from lycopene metabolism [56]. These cleavage products were 3-keto-apo-13-lycopenone and 3,4-dehydro-5,6-dihydro-15,15-apo-lycopenal, while the oxidation products were 2-apo-5,8-lycopenal-furanoxide, lycopene-5,6,5’,6’-diepoxide, lycopene-5,8-furanoxide isomer (I), lycopene-5,8-furanoxide isomer (II), and 3-keto-lycopene-5,8-furanoxide (Figure 2). An *in vitro* study using liposomal suspension showed that 8 carbonyl compounds namely 3, 7, 11-trimethyl-2, 4, 6, 10-dodecatetraen-1-al, 6, 10, 14-trimethyl-3, 5, 7, 9, 13 pentadecapentaen-2-one, acycloretinal, apo-14′-lycopenal, apo-12′-lycopenal, apo-10′-lycopenal, apo-8′-lycopenal, apo-6′-lycopenal and acycloretinoic acid were formed from lycopene oxidation [57]. In rats, 2 cleavage products were detected in the liver, which are apo-8′-lycopenal and apo-12´-lycopenal. However, Hu *et al.* [58] reported only apo-10′-lycopenal was found in ferret carotene-9',10'-monooxygenase catalyzed cleavage of carotenoids.

## 4. Lycopene as Antioxidant and Its Mechanism of Function

The reactivity of carotenoids, especially lycopene, in biological systems depends on their molecular and physical structure, location or site of action within the cells, ability to interact with other antioxidants, concentration and the partial pressure of oxygen [59,60]. Biologically, lycopene tends to act as singlet oxygen (^1^O_2_) and peroxyl radical scavenger (LOO•) [41]. Lycopene degradation may result in color loss when exposed to free radicals or oxidizing agents. This is due to the reaction with free radicals and causes interruption of the polyene chain, in which the conjugated double bond system may either be affected by cleavage or addition to one of the double bonds [26]. 

The highly conjugated double bonds of lycopene play the most important role in energy transfer reactions [60,61]. Lycopene has quenching ability towards singlet oxygen (^1^O_2_), based on the excited energy state, and is greatly related to the length of the conjugated double bond system [60]. Among the carotenoids, lycopene is the most efficient singlet oxygen quencher [62,63]. The physical quenching rate of lycopene was two times higher than β-carotene and 10 times higher than α-tocopherol [62]. 

**Figure 3:**
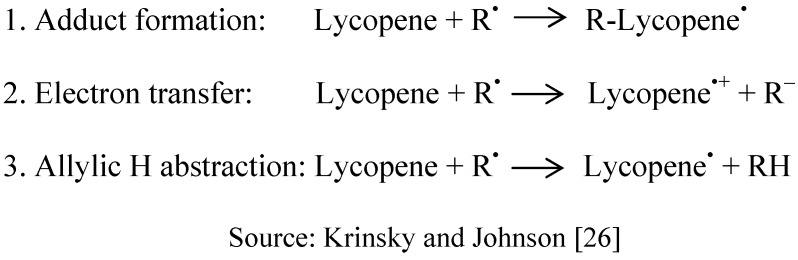
Three Possible Reactions of Carotenoids with Radical Species (R^•^).

Basically, chain lipid autoxidation reactions can be interrupted by antioxidants such as phenols, vitamin E and flavonoids, which eliminate the lipid peroxyl radicals by donating the hydrogen atom to form lipid peroxide and a resonance-stabilized antioxidant radical [64]. However, as a carotenoid compound, lycopene may scavenge the radicals by other ways. The mechanism of action for lycopene towards the reactive species can be predicted through three possible mechanisms: (1) adduct formation, (2) electron transfer to the radical and (3) allylic hydrogen abstraction [26,60,64,65,66], and is also shown in Figure 3. 

Adduct formation is the formation of resonance-stabilized carbon centered-peroxyl radicals where the free radical will attach to the polyene chain, the highly conjugated double bonds of lycopene, to form a lycopene-peroxyl radical adduct (ROO-lycopene^•^) [64,67]. This reaction is described in (1) where the lipid peroxyl radical (ROO^•^) reacts with lycopene.

(1) Lycopene + ROO^•^ 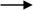 ROO-lycopene^•^

Under high oxygen concentrations, the ROO-lycopene^•^may possibly react with O_2_ to form a new radical (reaction 2). This reaction was reported as reversible and related to the pro-oxidant effect which may occur in carotenoid compounds [66]. 

(2) ROO-lycopene^•^ + O_2_ 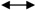 ROO-lycopene-OO^•^

The pro-oxidant effect of the peroxyl radical-lycopene adduct (ROO-lycopene^•^) can be explained if this compound is further reacted with oxygen forming a new lycopene-peroxyl radical (ROO-lycopene-OO^•^) [68]. This intermediate species (ROO-lycopene-OO^•^) will subsequently act as a pro-oxidant or initiator for lipid peroxidation by reacting with lipid (RH) (reaction 3) and forming another peroxyl radical (ROO^•^) with oxygen (O_2_) (reaction 4). 

(3) ROO-lycopene-OO^• ^+ RH 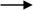 ROO-lycopene-OOH + R^•^

(4) R^• ^+ O_2_ 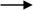 ROO^•^

However, the peroxyl radical-lycopene adduct may also be terminated in the occurence of another peroxyl radical by forming the inactive end products (reaction 5) [68].

(5) ROO-lycopene^•^ + ROO^• ^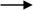 inactive products

Lycopene is one of the carotenoids prone to oxidation [65]. It is the best antioxidant based on electron transfer reactions [69]. Electron transfer, is the reaction with formation of carotenoid radicals such as lycopene cation radical (lycopene^+•^), anion radical (lycopene^-•^) or alkyl radical (lycopene^•^). Nitrogen dioxide radical (NO_2_^•^) from smoking, an environmental pollutant and the powerful oxidant trichloromethylperoxyl (CCl_3_O_2_^•^) may convert lycopene into radical cations (reaction 6 and 7) [65].

(6) NO_2_^• ^+ Lycopene 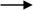 NO_2_^- ^+ Lycopene^+•^

(7) CCl_3_O_2_^• ^+ Lycopene 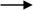 [CCl_3_O_2_- Lycopene]^• ^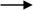 CCl_3_O_2_^- ^+ Lycopene^+•^

In addition, the reaction between lycopene and superoxide radical (O_2_^-•^) through electron transfer can form the lycopene anion radical (reaction 8) [70].

Lycopene + O_2_^-•^ 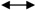 Lycopene ^-•^ + O_2_

However, hydrogen abstraction is the reaction of carotenoids as hydrogen donor to reduce the radical. The reaction is presented in reaction 9 [66].

(9) Lycopene + ROO^• ^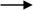 Lycopene^• ^+ ROOH

Again, the modes of action for antioxidants were depended on their position in the cell [60]. Carotenes such as lycopene lie parallelly with the membrane surface [71,72]. Thus, lycopene is expected to be a poor antioxidant due to its limited interaction with aqueous phase radicals in the lipid bilayer as compared to more polar carotenoids such as zeaxanthin [60]. Besides, high concentration of lycopene in the membranes may cause aggregation that may affect the properties of membrane by leading to increase in membrane fluidity and permeability, and finally will result in pro-oxidant type effects [73]. However, lycopene is still important in inhibiting lipid radicals at membranes as the first defense system of cells. Moreover, a combination of lycopene and other antioxidants is also important in scavenging of reactive species.

## 5. Interaction of Lycopene with Other Antioxidants

In lipid bilayer of cellular membrane, lycopene is expected to be a poor antioxidant due to its lesser interaction with aqueous phase radicals. However, the role of lycopene as a lipid phase antioxidant should not be neglected. The combinations of lycopene and other antioxidants such as vitamin C, vitamin E and β-carotene has exhibited higher scavenging activity on 2,2-diphenyl-1-picrylhydrazyl (DPPH) radical than their individual antioxidant activity [74]. Besides, lycopene combined with other antioxidants also gave a better inhibiting effect towards diene hydroperoxides produced from linoleic methyl ester with 2,2'-azobis (2,4-dimethylvaleronitrile) (AMVN) induced oxidation [75]. Lycopene was also reported to help in repairing the vitamin E radical (reaction 10) and the products from this reaction radical cation will be repaired by vitamin C (reactions 11 and 12) [76]. 

(10) Lycopene** + **TOH^+^**˙ **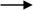 TOH + Lycopene^+^**˙**

(11) Lycopene^+^**˙ + **ASCH_2_ 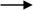 Lycopene + ASCH**˙ + **H^+^

(12) Lycopene^+^**˙ + **ASCH^-^ 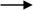 Lycopene + ASCH**˙**^-^ **+ **H^+^

Previously, lycopene was reported to react effectively with vitamin E radical in the lipophilic compartment [60]. Inversely, their reaction with the hydrophilic vitamin C was expected to be less effective. Yeum *et al.* [77] had suggested a model for the synergistic interactions among the antioxidants located in the hydrophilic and lipophilic compartments of plasma. Besides, there might be lycopene-carotenoid interaction in biological system (reaction 13). A study done using multilamellar liposomes showed that lycopene and lutein was the best combination toward AMVN-induced oxidation [78]. Lycopene is the strongest reducing agent and able to reduce the radical cations of lutein and zeaxanthin, but not β-carotene [79,80].

(13) Carotenoid^+^**˙ + **Lycopene 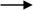 Carotenoid + Lycopene^+^**˙**

Different interpretations of reactions between lycopene with vitamin E and vitamin C is also reported [25,66,68]. Lycopene is suggested to protect tocopherol through the electron transfer to form α-tocopheroxyl radical (α-TO**˙**)(reaction 14) [81].

(14) α-TO**˙ **+ Lycopene 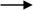 α-TOH + Lycopene^+^**˙**

On the other hand, some researchers suggestied that α-tocopherol (α-TOH) could reduce lycopene^+^**˙ **to regenerate the intact lycopene (reaction 15) [26].

(15) α -TOH + Lycopene^+^**˙ **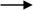 α-TO**˙** + Lycopene

However, a different reaction of lycopene radical cation (lycopene^+^**˙**) and α-tocopherol (α-TOH) or δ-tocopheroxyl radical (δ-TO**˙)** was also reported [82] as the following reactions (reactions 16 and 17).

(16) α -TOH + Lycopene^+^**˙ **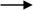 α-TO**˙ **+ Lycopene

(17) δ-TO**˙** + Lycopene 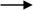 δ-TOH + Lycopene^+^**˙**

In non-polar solvents, carotenoids will probably react with α-tocopherol radical cation (α-TOH^+^**˙**) rather than with α-tocopherol anion (α-TOH**^-^**) as given in the reaction 18 [26]:

(18) α -TOH^+^**˙ **+ Lycopene 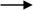 α-TOH**^-^** + Lycopene^+^**˙**

However, the reaction between lycopene and ascorbic acid increase the decay rate of Lycopene^+^**˙ **due to the following reaction (reaction 19) [26,66,83]:

(19) Lycopene^+^**˙**+ AscH**^-^** 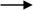 Lycopene + Asc**^-^˙**+ H^+^

Lycopene in combination with other antioxidants such as vitamins E and C, polyphenols and other carotenoids have wide potential for human health [60,84]. Recent formulations of antioxidant mixtures in the development of nutritional products has been in favour for their health benefits [85]. 

## 6. Preventive Effect of Lycopene toward Diseases

The effects of lycopene towards various diseases have been previously reviewed by many researchers. The protective effects of lycopene have been shown on oxidative stress, cardiovascular disease, hypertension, atherosclerosis, cancers, diabetes and others. However, there are still no conclusive results reported due to the fact studies on the role of lycopene against these diseases is still ongoing. 

### 6.1. Oxidative stress

Oxidative stress is one of the major risk factors of chronic diseases [86]. Free radicals or oxidants are potential contributors leading to oxidative stress. *In vitro, ex vivo*, and *in vivo* studies have been carried out to demonstrate the effects of lycopene against oxidative stress. In this context, lipid, protein and DNA oxidation are closely related to oxidative stress. 

Previous studies have reported lycopene-rich diet and lycopene supplementation provided protective effects against DNA damage in both normal and cancerous human cells [87,88,89]. In animals, reduction of lipid peroxidation products (thiobarbituric acid reactive substances, TBARS) and DNA damage markers were found in monkey kidney fibroblast and rat hepatocytes supplemented with lycopene (20 pmol/106 cells and 1.86–18.62 μM, respectively) [90,91]. Rats injected with lycopene (10 mg/kg/day, five days) also showed protective effect from iron-induced oxidative damage in prostate tissue and reduction of lipid peroxidation [92]. 

Human plasma lycopene levels have shown an inverse association with oxidative DNA damage [93]. Consumption of lycopene rich foods, juices or supplements has demonstrated protective effects against DNA damage in lymphocytes [47,94,95]. Besides, a high protection of lymphocytes from oxidative damage due to singlet oxygen and nitrogen dioxide was found in human subjects with the higher intake of lycopene-rich tomato juice [96]. Lycopene can protect human lymphoid cells from singlet oxygen by binding to the surface of the cells [97], but although consumption of tomato products has contributed to protecting lymphocytes from DNA damage, for lipid oxidation, a decrease in malondialdehyde (MDA) level was not found [98]. However, Riso *et al.* [99] reported no significant differences in endogenous lymphocyte DNA damage and 8-iso-prostaglandin F2α between a tomato-based drink treated group and placebo. 

A decrease of lipid and protein oxidation was also obtained in human consumed lycopene in the form of ketchup or oleoresin capsules [100,101]. Besides, the LDL oxidation and urinary 8-iso-prostaglandin F2α was found to be lower after the consumption of tomato products (8 mg lycopene/day, three weeks) [102]. Lycopene capsule supplementation (4 mg/day for six months) could substitute the hormone replacement therapy in postmenopausal women to prevent the oxidative stress and atherosclerosis [103]. Nevertheless, synergistic effect of lycopene with others antioxidants can be found. Tomato juice fortified with vitamin C gave a higher antioxidant capacity in urine and lower TBARS in plasma and urine [104].

### 6.2. Cardiovascular disease and related diseases

Cardiovascular disease (CVD) affects the normal function of the cardiovascular system involving heart and blood vessels. The World Health Organization (WHO) [105] reported that CVD is the world’s largest killer, claiming 17.1 million lives a year. Tobacco use, unhealthy diet, physical inactivity and high intake of alcohol increase the risk of CVD. Plasma low density lipoprotein (LDL) is the major risk factor of CVD. Increase in LDL oxidation is hypothesized to be causally associated with increasing risk of atherosclerosis and coronary heart disease. 

Study has shown that dietary lycopene supplementation (once a day, 1 week each) provided through tomato juice (50.4 mg lycopene), spaghetti sauce (39.2 mg lycopene) and tomato oleoresin (75.0 mg lycopene) will significantly increase serum lycopene [25]. Their study also showed that serum lipid peroxidation and LDL oxidation, significantly decreased after consuming lycopene rich foods, even though no difference was found in serum cholesterol levels. Besides, a high plasma level of lycopene was associated with a decreased risk of CVD in women [106]. 

The circulating plasma lycopene has been thought to prevent the development of atherosclerosis, especially in smokers [107]. Moreover, Rissanen *et al.* [108] suggested that serum lycopene may play an important role in the early stages of atherosclerosis. It also significantly reduced the formation of atherosclerotic plaques in the aorta and improved lipid profiles in high-fat diet rabbit compared to control group [109]. On the other hand, short term treatment of antioxidant rich tomato extract (250 mg/day, eight weeks) can reduce blood pressure in patients with hypertension [110]. A study by Rissanen *et al.* [111] exhibited an inverse relation between lycopene and intima-media thickness of the carotid artery as the risk factor for CVD. 

Nevertheless, there was a study reporting that a high serum level of total carotenes significantly decreased the risk of CVD mortality, but the inverse association between serum lycopene and risk of stroke mortality was not observed [112]. The fact is further supported by Osganian *et al.* [113] that significant inverse association was observed in α-carotene and β-carotene supplementation toward the risk of coronary artery disease, but no significant relation with intake of lycopene. Besides, dietary lycopene was not strongly associated with the decreased risk of CVD. Furthermore, Sesso *et al.* [114] suggested that dietary lycopene may confer preventive benefits on the cardiovascular system, but this possible association is significant for increasing the intake of tomato-based products such as tomato sauce and pizza.

### 6.3. Cancers

Cancer has emerged as a major public health problem around the world. This health issue has raised the awareness of people to go for natural products and their therapeutic or preventive value. The beneficial effect of lycopene is associated to decrease cancer incidence worldwide especially in prostate. Lycopene (1–4 µM) was also reported to reduce the risk of prostate, lung, leukemic and digestive tract cancers [115]. Besides, research was done on chemopreventive effects of lycopene (10–50 µM) in liver and ovary cells [89,116]. Although, there are chemopreventive effect but the lycopene concentrations used may exceed the normal biological available level.

Study has reported higher plasma lycopene were inversely associated with prostate cancer risk [117]. Lycopene was able to delay high-grade prostate intraepithelial neoplasia (HGPIN) from developing into prostate cancer and also inversely related to the prostate specific antigen [118]. Besides, lycopene (20–60 µM) was able to inhibit the proliferation of prostate cancer cells [119,120]. The antioxidative properties of lycopene was significantly diminished the DNA damage in prostate tissues [121]. Protective effects were also achieved with increase consumption of lycopene-rich diet [121,122]. According to Giovannucci *et al.* [123], frequent intake of tomato or lycopene was associated with lower risk of prostate cancer. 

In contrast, some studies showed no beneficial effects of lycopene intake. Kirsh *et al.* [124] reported lycopene, tomatoes or tomato-based foods intake was not associated with prostate cancer risk. Another study prescribed tomato supplement with lycopene (15 mg twice daily) to 46 patients with androgen-independent prostate cancer but the supplementation was not effective [125]. Case-control studies also showed no association between plasma lycopene on prostate cancer risk [126,127]. A synergistic effect of tea and lycopene on prostate cancer risk was also found if increase consumption of green tea with lycopene-rich fruits and vegetables [128]. Thus, it is important in consuming different bioactive compounds for better health instead of a single compound.

On the other hand, lycopene (1–10 µM) was able to inhibit human liver cancerous cells proliferation and preventing them from metastatic process [129,130]. Lycopene has significantly inversed the proliferation of human colon carcinoma, chronic lymphocytic leukemia, erythroleukemia and Burkitt lymphoma cell lines [115]. However, no anti-proliferation effect was found in lycopene treated skin carcinoma, prostate carcinoma, lung carcinoma, and breast carcinoma [130]. Even though, the anti-proliferative effect was dose dependent, Burgess *et al.* [130] mentioned that the effectiveness of lycopene in inhibiting the cancer cell lines needed an extra attention for its physiological achievable level as in human plasma (1–2 µM) and tissues (0.15–21.36 nmol/g tissue) [6,46]. However, the data on highest achievable level of lycopene concentration in human tissues with increasing lycopene intake is unknown. Besides, lycopene did not exhibit any efficiency in inhibiting the progression of preneoplastic oral lesions in hamster buccal pouch [131].

Study has shown that there was no association between some carotenoids and breast cancer among Chinese women, but increased intake of lycopene is associated to a reduced risk of breast cancer [132]. A cohort study concluded that neither high dietary nor plasma lycopene levels were associated with a reduced risk of breast cancer in middle-aged and older women [133]. Besides, no significantassociations were found between lycopene intake and lower risk of gastric cancer [134]. Moreover, apo-10'-lycopenoic acid (a compound converted from biological metabolite of lycopene) has demonstrated an inhibition effect towards lung cancer and suppression of lung tumor genesis in mice [135]. The beneficial effect of lycopene may be specific for certain organs. In mice study, mutagenesis in mice prostate was slightly inhibited by feeding lycopene-rich tomato oleoresin but mutagenesis was enhanced in the colon and lung [136].

### 6.4. Diabetes

Lycopene is closely related to various metabolic complications, especially diabetes. Serum lycopene is inversely associated with type-2 diabetes and impaired glucose metabolism. The fact is proven by Coyne *et al.* [137] that plasma glucose and fasting insulin concentrations decreased significantly with increase in serum lycopene. Besides, Polidori *et al.* [138] found that plasma lycopene were significantly lower in very old diabetic patients as compared to controls, while significant inverse correlations were found between age and lycopene.

In man, dietary lycopene was directly related to baseline serum concentrations of nonesterified fatty acids [139]. Besides that, there is also a concern about dietary lycopene and modulation of insulin-like growth factor (IGF). Riso *et al.* [140] have evaluated the effect of tomato drink intervention providing small amounts of lycopene and other carotenoids on serum levels of IGF-1. The results indicated that lycopene supplementation before and after each experimental period were inversely and significantly correlated with those of IGF-1. However, Wang *et al.* [141] have found low evidence for an association between baseline plasma lycopene and the risk of type-2 diabetes in middle-aged and older women after adjustment for multiple risk factors. 

### 6.5. Other health benefits and safety aspect

Lycopene has its ability to scavenge free radical. Thus lycopene may have health benefit effects and improvement of other disease conditions. Treatment of lycopene (1, 2 and 4 mg/kg; p.o.) in streptozotocin-induced diabetic rats has significantly attenuated cognitive deficit, increased acetylcholinesterase activity, oxidative-nitrosative stress and inflammation [142]. 

The treatment of lycopene using 3-nitropropionic acid-induced rats has significantly improved the memory and restored glutathione system functioning [143]. Akbaraly *et al.* [144] also suggested that low plasma lycopene levels could contribute to cognitive impairment. The list of lycopene effect on improvement of other disease impairments is shown in Table 3. 

**Table 3:** Action of lycopene in improving the impairment of other diseases.

Lycopene doses	Method	Impairment	Improvement	Lit. cited
0.2 mg/kg b.w. daily	*In vivo*–rats	Cataract	Significant delayed in the onset and progression of galactose cataract and reduced the incidence of selenite cataract.	145
2.5, 5 and 10 mg/kg b.w. daily	*In vivo*–rats	Cognitive function	Significant improved in memory.	143
60 mg/kg b.w. daily	*In vivo*–hyperlipemia rabbits	Lipid peroxidation injury	Significant reduced in the levels of serum TG and MDA, increase serum SOD activity, increase serum NO.	146
0.1, 0.5, 1, 2 g/kg b.w. daily	*In vivo*– mouse ear oedema model	Swelling	Decreased swelling of the croton oil-induced ear.	147
0, 5 and 10 μg/mLcarried by liposomes	*In vitro*–Calu-3 cells	Inflammation of cells infected by rhinovirus or exposed to lipopolysaccharide	Reduced the release of interleukin-6 and interferon-gamma induced protein-10.	148
8 or 16 mg/kg/day by i.p. injection	*In vivo*–murine model of asthma	Ovalbumin-induced inflammation	Significant inhibition of the infiltration of inflammatory immunocytes into the bronchoalveolar lavage.	149
2 mgtwice daily	*In vivo*–primigravida women	Pre-eclampsia and intrauterinegrowth retardation	Significant reduced in pre-eclamsia incidence and intrauterine growth retardation in the lycopene group compare to placebo group.	150
9 mg/kg b.w. twice a day for 2 weeks	*In vivo*–rats	Chronic bacterial prostatitis	Significant decreased in bacterial growth and improvement of prostatic inflammation.	151
0.025–2 mg per 20 mg b.w.	*In vivo*–white heterozygote mouse	X-ray radiation lesions	Moderate curative effect on the radiation lesions and increased survival rate	152

Lycopene supplementation in enucleated rat lenses culture has significantly (*p* < 0.001) restored glutathione and malondialdehyde levels, superoxide dismutase (*p* < 0.05), catalase and glutathione *S*-transferase (*p* < 0.01) [145]. However, no effect was found on glutathione peroxidase in the lycopene-supplemented group. Moreover, serum lycopene concentration was significantly lower in asthmatic [153] and subjects’ rheumatoid arthritis [154] than the control group. Dietary supplementation or adequate intake of lycopene and vitamin A rich foods may therefore be beneficial in asthmatic and rheumatoid arthritis.

The safety aspect of bioactive compounds in products has been received much attention from food scientists to avoid any side effects. Either synthetic lycopene or from natural sources have been reported to be safe (Generally Recognized as Safe, GRAS) when used in as food additive [155]. Toxicity studies have demonstrated that usage of synthetic lycopene in rats and rabbits will cause a direct maternal or developmental toxicity at high dosages 2 or 3 g/kg/day [156]. Hence, the safest observed level for lycopene intake is up to 75 mg/day [157]. Thrumbo [155] reviewed no adverse effects were found from animal consumption of dietary or formulated lycopene up to 3g/kg per day. However, only 7–10% of lycopene will be absorbed and 50% of it will be excreted through the feces and urine and the rest remains in the body [155].

## 7. Lycopene-Rich By-Products from Food Processing

Food processing by-products from the tomato puree and sauce industry are commonly used in the development of lycopene-rich products (Table 4). Previously, Al-Wandawi *et al.* [158] had reported that tomato skins contained a high amount of lycopene. Food processing waste is commonly used as feed for livestock. Among the agro-industrial by-products (cereal and pulsed, distillery, oil-seeds, sugar industry, textile industry, vegetables and fruits industry, vegetables crop, and miscellancous), tomato wastes are the only by-products that are rich in lycopene [159]. 

**Table 4:** Studies on lycopene from by-products.

Country	By-products	References
Algeria	Tomato skin	160
Argentina	Tomato skin	161
Canada	Tomato skin	162
China, Canada	Tomato paste waste	163
China	Tomato paste waste	164
	Tomato paste waste	165
India	Mace (*Myristica fragrans*)	166
	Tomato peels and seeds, tomato industrial waste	167
	Tomato skin	20
Iraq	Tomato skin	158
Italy	Tomato peels and seeds	168
	Tomato peels	169
Hungary	Tomato pomace	19
Japan	Tomato skin	18
Portugal, Brazil	Tomato skin and seeds	170
Spain	Tomato peels	171
Taiwan	Tomato pulp waste	172
Turkey, Netherland	Tomato paste waste	173
USA	Tomato pomace	174

Nowadays, there is an increasing trend towards utilization of food processing by-products as a source of functional components [175]. Many studies have been carried out on the extraction of lycopene from by-products especially tomato waste. Optimization of the solvent extraction procedure was also performed to obtain a maximum lycopene yield from tomato peels using response surface methodology [20]. Application of high hydrostatic pressure processing without heating was reported to provide an increased yield of lycopene from tomato paste waste [165]. High pressure processing of tomato paste waste for 1 min gives a higher lycopene yield than solvent extraction for 30 min [164]. The Extractor Naviglio has been introduced to obtain higher purity lycopene from tomato by-products through pressurized extraction [161,176]. This extraction method requires tap water as extracting solvent with minimum organic solvent and the by-products can be further used as livestock feed. Furthermore, enzymatic treatment using cellulase and pectinase could offer one fold higher in the recovery of lycopene from tomato waste [167]. Lavecchia and Zuorro [169] also reported that enzymatic treatment on tomato peels was able to increase the lycopene yield 20-fold. Moreover, supercritical fluid extraction has been applied in extraction of lycopene from several by-products [17,19,170]. Optimization of different extraction parameters on lycopene-rich by-products using supercritical fluid extraction were also studied [18,162,168,173]. Supercritical fluid extract of lycopene-rich tomato pulp waste has been used for encapsulation using an emulsion system in combination with gelatin and poly (γ-glutamic acid) (γ-PGA) as coating materials [172].

On the other hand, there are initiatives by food scientist to recycle the lycopene-rich by-products as food ingredients. Fortification with lycopene in dry fermented sausage was also done by adding dried tomato peel to the meat mixture during the sausage production [171]. The development of extrusion processing using barley-tomato pomace blends and processing into snacks has been demonstrated by Altan *et al.* [174]. Besides, enrichment of low quality edible oils such as refined olive oil, extra virgin olive oil and refined sunflower oil by lycopene from tomato peels or tomato puree was proven to induce thermal stability to these edible oils [160]. The idea of using lycopene-rich by-products from tomato peel and seed for hen feed will further enrich the egg yolk with lycopene. However, only low amounts of lycopene were found to be transferred to the egg yolk (0.1% from tomato peels and 0.7% from tomato seeds) [177]. Another study also determined the quality of lycopene-rich by-products after food processing such as blanching and drying, where blanching in hot water at 75 °C for 2 min could help to reduce the drying time and increase the lycopene bio-availability [166].

## 8. Thermal Process on Lycopene Content

Thermal processing is used in the food industry to preserve food products and maintain the nutritional quality. Traditionally, sun drying is the easiest and cheapest technique, and it is commonly used in poor countries or small and medium industries for food preservation. However the disadvantages are food processing enhances lycopene destruction and increases the process duration [3]. The alternative method is oven drying of the food materials. However, lycopene is a heat sensitive compound and degraded when exposed to heat. The temperature is an important factor for thermal processing in order to remove the moisture with minimum destruction of lycopene and other nutrients. Besides, heating of lycopene in oil bath at different times had been shown to enhance the degradation of lycopene when increased in temperature from 50 to 150 °C [178].

Chang *et al.* [179] reported that thermal processing enhanced lycopene isomerization and increased lycopene extracting ability by breaking down the cell walls and weakening the interaction between lycopene and the tissue matrix of samples. Hot air drying at 80 °C for the first 2 h plus shifting the drying temperature to 60 °C for another 6 h were reported to yield higher lycopene content as compared to fresh and freeze dried sample [179]. Besides, treatment of tomatoes with forced air drying at 42 °C for 48 h has shown a significant increase in lycopene contents [180]. In contrast, semi-drying method for drying of tomatoes using a forced air drying at 42 °C for 8 h showed a significant decrease in lycopene content [181].

Lycopene stability is always considered by researchers to ensure that lycopene is able to be preserved until utilization. A study done by Shi *et al.* [182] showed that higher levels of lycopene *cis* isomers, lower total lycopene and *trans* isomers were obtained from tomato using air drying method at 95 °C for 6–10 h as compared to the vacuum drying and osmotic treatment methods. However, lycopene and other lipophilic antioxidant compounds in tomato pulps have high stability after air drying [183].

In air drying processing, total lycopenes was affected by isomerization and oxidation, while there was a significant increase in *cis* isomers and decreased in *trans* isomers when the temperature and processing time increased [182]. Thus, the duration of thermal process also play an important role in lycopene accessibility analysis. Besides, Hsu [184] revealed that hot-break processing (92 °C for 2 min) and cold-break processing (60 °C for 2 min) did not enhance the lycopene extractability and degradation. It was probably due to insufficient temperature and time.

Moisture content is closely related to lycopene degradation. When moisture is retained, the water soluble compounds will react as catalyst during lycopene degradation. Goula *et al.* [185] reported that degradation of lycopene in tomato pulp was reduced when the moisture content decreased from 95% to 55%, with a minimum degradation rate in between 50 to 55% of moisture content. Thus, the catalytic effect of lycopene degradation will eliminated when the moisture is removed. 

## 9. Conclusions

Lycopene is the red pigment that plays an important role in plant and animals. In human health, much evidence shows that consumption of lycopene rich foods can help in preventing degenerative diseases, but very limited studies have found a beneficial role of the consumption of lycopene alone. The interaction of lycopene with other active compounds is crucial for obtaining its optimal function in human health. On the other hand, some beneficial effects may due to the lycopene isomers or its metabolites but information about this is scarce. In addition, further studies on the biological activity of lycopene or with the combination of different compounds are also warranted. Besides, there are only limited studies on long term intake of lycopene that might provide information about the upper limit of lycopene intake. The development of clean technology for high quality nutraceutical products is also needed for the promotion of lycopene consumption. Nonetheless, further understanding of the clinical aspects of lycopene, its mechanism of action towards diseases, bioavailability, bioaccessibility, recommended intake, interaction with other compounds and its metabolites activities are needed due to the lack of conclusive results on the role of lycopene in human health.

## References

[1] Mortensen A. (2006). Carotenoids and other pigments as natural colorants. Pure Appl. Chem..

[2] Vogele A.C. (1937). Effect of environmental factors upon the color of the tomato and the watermelon. Plant Physiol..

[3] Rodriguez-Amaya D.B., Kimura M. (2004). Carotenoids in foods. Harvestplus Handbook for Carotenoid Analysis.

[4] Rodriguez-Amaya D.B. (2001). A Guide to Carotenoid Analysis in Foods.

[5] (2007). Focus on Pigments. World spends more than $50 M on lycopene red. Focus Pigm..

[6] Rao A.V., Argawal S. (1999). Role of lycopene as antioxidant carotenoid in the prevention of chronic diseases: A review. Nutr. Res..

[7] Scordino M., Di Mauro A., Passerini A., Maccarone E. (2005). Selective recovery of anthocyanins and hydroxycinnamates from a byproduct of citrus processing. J. Agric. Food Chem..

[8] Amin I., Mukhrizah O. (2006). Antioxidant capacity of methanolic and water extracts prepared from food-processing by-products. J. Sci. Food Agric..

[9] Ajila C.M., Naidu K.A., Bhat S.G., Prasada Rao U.J.S. (2007). Bioactive compounds and antioxidant potential of mango peel extract. Food Chem..

[10] Abdalla A.E.M., Darwish S.M., Ayad E.H.E., El-Hamahmy R.M. (2007). Egyptian mango by-product 2: Antioxidant and antimicrobial activities of extract and oil from mango seed kernel. Food Chem..

[11] Correia R.T.P., Mccue P., Magalhães M.M.A., Macêdo G.R., Shetty K. (2004). Phenolic antioxidant enrichment of soy flour supplemented guava waste by rizhopus oligosporus-mediated solid-state bioprocessing. J. Food Biochem..

[12] Bernardino-Nicanor A., Anón M.C., Scilingo A.A., Dávila-Ortíz G. (2005). Functional Properties of Guava Seed Glutelins. Journal of Agricultural and Food Chem..

[13] Thongsombat W., Sirichote A., Chanthachum S. (2007). The production of guava juice fortified with dietary fiber. Songklanakarin J. Sci. Technol..

[14] Kong K.W., Ismail A., Tan C.P., Rajab N.F. (2009). Optimization of oven drying conditions for lycopene content and lipophilic antioxidant capacity in a by-product of the pink guava puree industry using response surface methodology. LWT-Food Sci. Technol..

[15] Li Y., Guo C., Yang J., Wei J., Xu J., Cheng S. (2006). Evaluation of antioxidant properties of pomegranate peel extract in comparison with pomegranate pulp extract. Food Chem..

[16] Hajimahmoodi M., Oveisi M.R., Sadeghi N., Jannat B., Hadjibabaie M., Farahani E., Akrami M.R., Namdar R. (2008). Antioxidant properties of peel and pulp hydro extract in ten Persian pomegranate cultivars. Pak. J. Biol. Sci..

[17] Rozzi N.L., Singh R.K., Vierling R.A., Watkins B.A. (2002). Supercritical fluid extraction of lycopene from tomato processing byproducts. J. Agric.Food Chem..

[18] Topal U., Sasaki M., Goto M., Hayakawa K. (2006). Extraction of lycopene from tomato skin with supercritical carbon dioxide: effect of operating conditions and solubility analysis. J. Agric.Food Chem..

[19] Vagi E., Simandi B., Vasarhelyine K.P., Daood H., Kery A., Doleschall F., Nagy B. (2007). Supercritical carbon dioxide extraction of carotenoids, tocopherols and sitosterols from industrial tomato by-products. J. Supercrit. Fluids.

[20] Kaur D., Wani A.A., Oberoi D.P.S., Sogi D.S. (2008). Effect of extraction conditions on lycopene extractions from tomato processing waste skin using response surface methodology. Food Chem..

[21] Chantaro P., Devahastin S., Chiewchan N. (2008). Production of antioxidant high dietary fiber powder from carrot peels. LWT-Food Sci. Technol..

[22] Stah W., Sies H. (1996). Lycopene: A biologically important carotenoid for humans?. Arch. Biochem. Biophys..

[23] Shi J., Le Maguer M., Bryan M., Shi J., Mazza G., Le Maguer M. (2002). Lycopene from tomatoes. Functional Foods-Biochemical and Processing Aspects.

[24] Roldán-Gutiérrez J.M., Dolores Luque de Castro M. (2007). Lycopene: The need for better methods for characterization and determination. Trends Anal. Chem..

[25] Agarwal S., Rao A.V. (1998). Tomato Lycopene and Low Density Lipoprotein Oxidation: A Human Dietary Intervention Study. Lipids.

[26] Krinsky N.I., Johnson E.J. (2005). Carotenoid actions and their relation to health and disease. Mol. Aspects Med..

[27] Clinton S.K. (1998). Lycopene: chemistry, biology, and implications for human health and disease. Nutr. Rev..

[28] During A., Harrison E.H. (2004). Intestinal absorption and metabolism of carotenoids: Insights from cell culture. Arch. Biochem. Biophys..

[29] During A., Harrison E.H. (2005). An *in vitro* model to study the intestinal absorption of carotenoids. Food Res. Int..

[30] Goñi I., Serrano J., Saura-Calixto F. (2006). Bioaccessibility of β-carotene, lutein, and lycopene from fruits and vegetables. J. Agric. Food Chem..

[31] Riedl J., Linseisen J., Hoffmann J., Wolfram G. (1999). Some dietary fibers reduce the absorption of carotenoids in women. J. Nutr..

[32] Failla M.L., Chitchumroonchokchai C., Ishida B.K. (2008). In vitro micellarization and intestinal cell uptake of cis isomers of lycopene exceed those of all-trans lycopene. J. Nutr..

[33] Richelle M., Bortlik K., Liardet S., Hager C., Lambelet P., Baur M., Applegate L.A., Offord E.A. (2002). A food-based formulation provides lycopene with the same bioavailability to humans as that from tomato paste. J. Nutr..

[34] Re R., Fraser P.D., Long M., Bramley P.M., Rice-Evans C. (2001). Isomerization of lycopene in the gastric milieu. Biochem. Biophys. Res. Commun..

[35] Unlu N.Z., Bohn T., Francis D.M., Nagaraja H.N., Clinton S.K., Schwartz S.J. (2007). Lycopene from heat-induced cis-isomer-rich tomato sauce is more bioavailable than from all-trans-rich tomato sauce in human subjects. Br. J. Nutr..

[36] Karakaya S., Yilmaz N. (2007). Lycopene content and antioxidant activity of fresh and processed tomatoes and in vitro bioavailability of lycopene. J. Sci. Food Agric..

[37] Ahuja K.D.K., Pittaway J.K., Ball M.J. (2006). Effects of olive oil and tomato lycopene combination on serum lycopene, lipid profile, and lipid oxidation. Nutrition.

[38] Brown M.J., Ferruzzi M.G., Nguyen M.L., Cooper D.A., Eldridge A.L., Schwartz S.J., White W.S. (2004). Carotenoid bioavailability is higher from salads ingested with full-fat than with fat-reduced salad dressings as measured with electrochemical detection. Am. J. Clin. Nutr..

[39] Fielding J.M., Rowley K.G., Cooper P., O'Dea K. (2005). Increases in plasma lycopene concentration after consumption of tomatoes cooked with olive oil. Asia-Pac J. Clin. Nutr..

[40] Cardinault N., Tyssandier V., Grolier P., Winklhofer-Roob B.M., Ribalta J., Bouteloup-Demange C., Rock E., Borel P. (2003). Comparison of the postprandial chylomicron carotenoid responses in young and older subjects. Eur. J. Nutr..

[41] Stahl W., Sies H. (2003). Antioxidant activity of carotenoids. Mol. Aspects Med..

[42] Boileau T.W.-M., Boileau A.C., Erdman J.W. (2002). Bioavailability of all-trans and cis-isomers of lycopene. Exp. Bio. Med..

[43] Erdman J.W. (2005). How do nutritional and hormonal status modify the bioavailability, uptake, and distribution of different isomers of lycopene?. J. Nutr..

[44] Goralczyk R., Siler U., Bao Y., Fenwick R. (2004). The Role of Lycopene in Human Health. Phytochemicals in Health and Disease.

[45] Zaripheh S., Boileau T.W.-M., Lila M.A., Erdman J.W. Jr. (2003). [14C]-lycopene and [14C]-labeled polar products are differentially distributed in tissues of F344 rats prefed lycopene. J. Nutr..

[46] Su Q., Rowley K.G., Balazs N.D.H. (2002). Carotenoids: Separation methods applicable to biological samples. J. Chromatogr. B.

[47] Porrini M., Riso P., Brusamolino A., Berti C., Guarnieri S., Visioli F. (2005). Daily intake of a formulated tomato drink affects carotenoid plasma and lymphocyte concentrations and improves cellular antioxidant protection. Br. J. Nutr..

[48] Stimpson J.P., Lackan N.A. (2007). Serum carotenoid levels vary by marital status. J. Am. Dietetic Assoc..

[49] Scott K.J., Thurnham D.I., Hart D.J., Bingham S.A., Day K. (1996). The correlation between the intake of lutein, lycopene and beta-carotene from vegetables and fruits, and blood plasma concentrations in a group of women aged 50-65 years in the UK. Br. J. Nutr..

[50] Michaud D.S., Giovannucci E.L., Ascherio A., Rimm E.B., Forman M.R., Sampson L., Willett W.C. (1998). Associations of plasma carotenoid concentrations and dietary intake of specific carotenoids in samples of two prospective cohort studies using a new carotenoid database. Cancer Epidemiol. Biomarkers Prev..

[51] Olmedilla B., Granado F., Southon S., Wright A.J., Blanco I., Gil-Martinez E., Berg H., Corridan B., Roussel A.M., Chopra M., Thurnham D.I. (2001). Serum concentrations of carotenoids and vitamins A, E, and C in control subjects from five European countries. Br. J. Nutr..

[52] Al-Delaimyl W.K., Van Kappel A.L., Ferrari P., Slimani N., Steghens J.-P., Bingham S., Johansson I., Wallström P., Overvad K., Tjønneland A., Key T.J., Welch A.A., Bueno-de-Mesquita H.B., Peeters P.H.M., Boeing H., Linseisen J., Clavel-Chapelon F., Guibout C., Navarro C., Quirós J.R., Palli D., Celentano E., Trichopoulou A., Benetou V., Kaaks R., Riboli E. (2004). Plasma levels of six carotenoids in nine European countries: Report from the European Prospective Investigation into Cancer and Nutrition (EPIC). Publ. Health Nutr..

[53] Ozasa K., Ito Y., Suzuki K., Watanabe Y., Wakai K., Tamakoshi A. (2005). Association of serum carotenoid concentration and dietary habits among the JACC Study subjects. J. Epidemiol..

[54] Boonsiri P., Pooart J., Tangrassameeprasert R., Hongsprabhas P. (2007). Serum β-carotene, lycopene and α-tocopherol levels of healthy people in northeast Thailand. Asia-Pac. J. Clin. Nutr..

[55] Lindshield B.L., Canene-Adams K., Erdman J.W. (2007). Lycopenoids: Are lycopene metabolites bioactive?. Arch. Biochem. Biophys..

[56] Ferreira A.L.D.A., Yeum K.-J., Russell R.M., Krinsky N.I., Tang G. (2003). Enzymatic and oxidative metabolites of lycopene. J. Nutr. Biochem..

[57] Kim S.-J., Nara E., Kobayashi H., Terao J., Nagao A. (2001). Formation of cleavage products by autoxidation of lycopene. Lipids.

[58] Hu K.Q., Liu C., Ernst H., Krinsky N.I., Russell R.M., Wang X.D. (2006). The biochemical characterization of ferret carotene-9',10'-monooxygenase catalyzing cleavage of carotenoids in vitro and in vivo. J. Biol. Chem..

[59] Britton G. (1995). Structure and properties of carotenoids in relation to function. FASEB J..

[60] Young A.J., Lowe G.M. (2001). Antioxidant and prooxidant properties of carotenoids. Arch. Biochem. Biophys..

[61] Christensen R.L., Frank H.A., Young A.J., Britton G., Cogdell R.J. (1999). The Photochemistry of Carotenoids.

[62] Di Mascio P., Kaiser S., Sies H. (1989). Lycopene as the most efficient biological carotenoid singlet oxygen quencher. Arch. Biochem. Biophys..

[63] Cantrell A., McGarvey D.J., Truscott T.G., Rancan F., Böhm F. (2003). Singlet oxygen quenching by dietary carotenoids in a model membrane environment. Arch. Biochem. Biophys..

[64] El-Agamey A., Lowe G.M., McGarvey D.J., Mortensen A., Phillip D.M., Truscott T.G., Young A.J. (2004). Carotenoid radical chemistry and antioxidant or pro-oxidant properties. Arch. Biochem. Biophys..

[65] Mortensen A., Skibsted L.H., Truscott T.G. (2001). The interaction of dietary carotenoids with radical species. Arch. Biochem. Biophys..

[66] Krinsky N.I., Yeum K.J. (2003). Carotenoid-radical interactions. Biochem. Biophys. Res. Commun..

[67] Burton G.W., Ingold K.U. (1984). β-Carotene: An unusual type of lipid antioxidant. Science.

[68] Kiokias S., Gordon M.H. (2004). Antioxidant properties of carotenoids in vitro and in vivo. Food Rev. Int..

[69] Mortensen A., Skibsted L.H. (1998). Reactivity of β-carotene towards peroxyl radicals studied by laser flash and steady-state photolysis. FEBS Lett..

[70] Conn P.F., Lambert C., Land E.J., Schalch W., Truscott T.G. (1992). Carotene-oxygen radical interactions. Free Radical Res. Commun..

[71] Johansson L.B., Lindblom G., Wieslander Å., Arvidson G. (1981). Orientation of β-carotene and retinal in lipid bilayers. FEBS Lett..

[72] Van de Ven M., Kattenberg M., van Ginkel G., Levine Y.K. (1984). Study of the orientational ordering of carotenoids in lipid bilayers by resonance-Raman spectroscopy. Biophys. J..

[73] Lowe G.M., Booth L.A., Young A.J., Bilton R.F. (1999). Lycopene and β-carotene protect against oxidative damage in HT29 cells at low concentrations but rapidly lose this capacity at higher doses. Free Radical Res..

[74] Liu D., Shi J., Colina Ibarra A., Kakuda Y., Jun Xue S. (2008). The scavenging capacity and synergistic effects of lycopene, vitamin E, vitamin C, and β-carotene mixtures on the DPPH free radical. LWT-Food Sci. Technol..

[75] Shi J., Qu Q., Kakuda Y., Xue S.J., Jiang Y., Koide S., Shim Y.-Y. (2007). Investigation of the antioxidant and synergistic activity of lycopene and other natural antioxidants using LAME and AMVN model systems. J. Food Compos. Anal..

[76] Truscott T.G. (1996). β-carotene and disease: A suggested pro-oxidant and anti-oxidant mechanism and speculations concerning its role in cigarette smoking. J. Photochem. Photobiol. B: Biol..

[77] Yeum K.-J., Russell R.M., Krinsky N.I., Aldini G. (2004). Biomarkers of antioxidant capacity in the hydrophilic and lipophilic compartments of human plasma. Arch. Biochem. Biophys..

[78] Stahl W., Junghans A., de Boer B., Driomina E.S., Briviba K., Sies H. (1998). Carotenoid mixtures protect multilamellar liposomes against oxidative damage: synergistic effects of lycopene and lutein. FEBS Lett..

[79] Edge R., Land E.J., McGarvey D., Mulroy L., Truscott T.G. (1998). Relative one electron reduction potentials of carotenoid radical cations and the interactions of carotenoids with the vitamin E radical cation. J. Am. Chem. Soc..

[80] Edge R., Truscott T.G., Frank H.A., Young A.J., Britton G., Cogdell R.J. (1999). The Photochemistry of Carotenoids.

[81] Böhm F., Edge R., Land E.J., McGarvey D.J., Truscott T.G. (1997). Carotenoids enhance vitamin E antioxidant efficiency. J. Am. Chem. Soc..

[82] Mortensen A., Skibsted L.H. (1997). Relative stability of carotenoid radical cations and homologue tocopheroxyl radicals: A real time kinetic study of antioxidant hierarchy. FEBS Lett..

[83] Truscott T.G., Walter P., Hornig D., Moser U. (2001). Synergistic effects of antioxidant vitamins. Functions of Vitamins beyond Recommended Dietary Allowances.

[84] Shixian Q., Dai Y., Kakuda Y., Shi J., Mittal G., Yeung D., Jiang Y. (2005). Synergistic anti-oxidative effects of lycopene with other bioactive compounds. Food Rev. Int..

[85] Castro I.A., Moraes Barros S.B., Lanfer Marquez U.M., Motizuki M., Higashi Sawada T.C. (2005). Optimization of the antioxidant capacity of a mixture of carotenoids and α-tocopherol in the development of a nutritional supplement. Food Res. Int..

[86] Rao A.V., Rao L.G. (2007). Carotenoids and human health. Pharmacol. Res..

[87] Astley S.B., Hughes D.A., Wright A.J.A., Elliott R.M., Southon S. (2004). DNA damage and susceptibility to oxidative damage in lymphocytes: effects of carotenoids *in vitro* and *in vivo*. Br. J. Nutr..

[88] Liu F., Zhang Z.-Z., Wu M., Shu Y. (2007). Study on the protective effect of tomato juice on DNA damage in cells. J. Sichuan Univ. (Medical Science Edition).

[89] Scolastici C., Alves de Lima R.O., Barbisan L.F., Ferreira A.L.A., Ribeiro D.A., Salvadori D.M.F. (2008). Antigenotoxicity and antimutagenicity of lycopene in HepG2 cell line evaluated by the comet assay and micronucleus test. Toxicol. in Vitro.

[90] Matos H.R., Di Mascio P., Medeiros M.H.G. (2000). Protective effect of lycopene on lipid peroxidation and oxidative DNA damage in cell culture. Arch. Biochem. Biophys..

[91] Srinivasan M., Sudheer A.R., Pillai K.R., Kumar P.R., Sudhakaran P.R., Menon V.P. (2007). Lycopene as a natural protector against γ-radiation induced DNA damage, lipid peroxidation and antioxidant status in primary culture of isolated rat hepatocytes *in vitro*. Biochim. Biophys. Acta-Gen. Sub..

[92] Matos H.R., Marques S.A., Gomes O.F., Silva A.A., Heimann J.C., Di Mascio P., Medeiros M.H.G. (2006). Lycopene and β-carotene protect in vivo iron-induced oxidative stress damage in rat prostate. Braz. J. Med. Biol. Res..

[93] Watters J.L., Satia J.A., Kupper L.L., Swenberg J.A., Schroeder J.C., Switzer B.R. (2007). Associations of antioxidant nutrients and oxidative DNA damage in healthy African-American and White adults. Cancer Epidemiol. Biomark. Prev..

[94] Riso P., Pinder A., Santangelo A., Porrini M. (1999). Does tomato consumption effectively increase the resistance of lymphocyte DNA to oxidative damage?. Am. J. Clin. Nutr..

[95] Zhao X., Aldini G., Johnson E.J., Rasmussen H., Kraemer K., Woolf H., Musaeus N., Krinsky N.I., Russell R.M., Yeum K.-J. (2006). Modification of lymphocyte DNA damage by carotenoid supplementation in postmenopausal women. Am. J. Clin. Nutr..

[96] Böhm F., Edge R., Burke M., Truscott T.G. (2001). Dietary uptake of lycopene protects human cells from singlet oxygen and nitrogen dioxide: ROS components from cigarette smoke. J. Photochem. Photobiol. B: Biol..

[97] Tinkler J.H., Bohm F., Schalch W., Truscott T.G. (1994). Dietary carotenoids protect human cells from damage. J. Photochem. Photobiol. B: Biol..

[98] Riso P., Visioli F., Erba D., Testolin G., Porrini M. (2004). Lycopene and vitamin C concentrations increase in plasma and lymphocytes after tomato intake. Effects on cellular antioxidant protection. Eur. J. Clin. Nutr..

[99] Riso P., Visioli F., Grande S., Guarnieri S., Gardana C., Simonetti P., Porrini M. (2006). Effect of a tomato-based drink on markers of inflammation, immunomodulation, and oxidative stress. J. Agric. Food Chem..

[100] Rao A.V., Shen H. (2002). Effect of low dose lycopene intake on lycopene bioavailability and oxidative stress. Nutr. Res..

[101] Rao L.G., Mackinnon E.S., Josse R.G., Murray T.M., Strauss A., Rao A.V. (2007). Lycopene consumption decreases oxidative stress and bone resorption markers in postmenopausal women. Osteoporos. Int..

[102] Visioli F., Riso P., Grande S., Galli C., Porrini M. (2003). Protective activity of tomato products on in vivo markers of lipid oxidation. Eur. J. Nutr..

[103] Misra R., Mangi S., Joshi S., Mittal S., Gupta S.K., Pandey R.M. (2006). LycoRed as an alternative to hormone replacement therapy in lowering serum lipids and oxidative stress markers: A randomized controlled clinical trial. J. Obstet. Gynaecol. Res..

[104] Jacob K., Periago M.J., Böhm V., Berruezo G.R. (2008). Influence of lycopene and vitamin C from tomato juice on biomarkers of oxidative stress and inflammation. Br. J. Nutr..

[105] WHO Cardiovascular Diseases: World Heart Day 2009. http://www.who.int/cardiovascular_diseases/en/,.

[106] Sesso H.D., Buring J.E., Norkus E.P., Gaziano J.M. (2004). Plasma lycopene, other carotenoids, and retinol and the risk of cardiovascular disease in wome. Am. J. Clin. Nutr..

[107] Klipstein-Grobusch K., Launer L.J., Geleijnse J.M., Boeing H., Hofman A., Witteman J.C.M. (2000). Serum carotenoids and atherosclerosis The Rotterdam Study. Atherosclerosis.

[108] Rissanen T.H., Voutilainen S., Nyyssönen K., Salonen J.T. (2002). Lycopene, atherosclerosis, and coronary heart disease. Exp. Biol. Med..

[109] Hu M.-Y., Li Y.-L., Jiang C.-H., Liu Z.-Q., Qu S.-L., Huang Y.-M. (2008). Comparison of lycopene and fluvastatin effects on atherosclerosis induced by a high-fat diet in rabbits. Nutrition.

[110] Engelhard Y.N., Gazer B., Paran E., Sheva B. (2006). Natural antioxidants from tomato extract reduce blood pressure in patients with grade-1 hypertension: A double-blind, placebo-controlled pilot study. Am. Heart J..

[111] Rissanen T.H., Voutilainen S., Nyyssönen K., Salonen R., Kaplan G.A., Salonen J.T. (2003). Serum lycopene concentrations and carotid atherosclerosis: the Kuopio Ischaemic Heart Disease Risk Factor Study. Am. J. Clin. Nutr..

[112] Ito Y., Kurata M., Suzuki K., Hamajima N., Hishida H., Aoki K. (2006). Cardiovascular Disease Mortality and Serum Carotenoid Levels: A Japanese Population-based Follow-up Study. J. Epidemiol..

[113] Osganian S.K., Stampfer M.J., Rimm E., Spiegelman D., Manson J.E., Willett W.C. (2003). Dietary carotenoids and risk of coronary artery disease in women. Am. J. Clin. Nutr..

[114] Sesso H.D., Liu S., Gaziano J.M., Burin J.E. (2003). Dietary Lycopene, Tomato-Based Food Products and Cardiovascular Disease in Women. J. Nutr..

[115] Salman H., Bergman M., Djaldetti M., Bessler H. (2007). Lycopene affects proliferation and apoptosis of four malignant cell lines. Biomed. Pharmacother..

[116] Scolastici C., Alves de Lima R.O., Barbisan L.F., Ferreira A.L.A., Ribeiro D.A., Salvadori D.M.F. (2007). Lycopene activity against chemically induced DNA damage in Chinese hamster ovary cells. Toxicol. in Vitro.

[117] Zhang J., Dhakal I., Stone A., Ning B., Greene G., Lang N.P., Kadlubar F.F. (2007). Plasma carotenoids and prostate cancer: A population-based case-control study in Arkansas. Nutr. Cancer.

[118] Mohanty N.K., Saxena S., Singh U.P., Goyal N.K., Arora R.P. (2005). Lycopene as a chemopreventive agent in the treatment of high-grade prostate intraepithelial neoplasia. Urol. Oncol.-Semin. Ori..

[119] Kanagaraj P., Vijayababu M.R., Ravisankar B., Anbalagan J., Aruldhas M.M., Arunakaran J. (2007). Effect of lycopene on insulin-like growth factor-I, IGF binding protein-3 and IGF type-I receptor in prostate cancer cells. J. Cancer Res. Clin. Oncology.

[120] Gunasekera R.S., Sewgobind K., Desai S., Dunn L., Black H.S., McKeehan W.L., Patil B. (2007). Lycopene and lutein inhibit proliferation in rat prostate carcinoma cells. Nutr. Cancer.

[121] Stacewicz-Sapuntzakis M., Bowen P.E. (2005). Role of lycopene and tomato products in prostate health. Biochim. Biophys. Acta-Mol. Basis Dis..

[122] Hadley C.W., Miller E.C., Schwartz S.J., Clinton S.K. (2002). Tomatoes, lycopene, and prostate cancer: Progress and promise. Exp. Biol. Med..

[123] Giovannucci E., Rimm E.B., Liu Y., Stampfer M.J., Willett W.C. (2002). A Prospective Study of Tomato Products, Lycopene, and Prostate Cancer Risk. J. Nat. Cancer Inst..

[124] Kirsh V.A., Mayne S.T., Peters U., Chatterjee N., Leitzmann M.F., Dixon L.B., Urban D.A., Crawford E.D., Hayes R.B. (2006). A prospective study of lycopene and tomato product intake and risk of prostate cancer. Cancer Epidemiol. Biomark. Prev..

[125] Jatoi A., Burch P., Hillman D., Vanyo J.M., Dakhil S., Nikcevich D., Rowland K., Morton R., Flynn P.J., Young C., Tan W. (2007). A tomato-based, lycopene-containing intervention for androgen-independent prostate cancer: Results of a phase II study from the North Central Cancer Treatment Group. Urology.

[126] Key T.J., Appleby P.N., Allen N.E., Travis R.C., Roddam A.W., Jenab M., Egevad L., Tjonneland A., Johnsen N.F., Overvad K., Linseisen J., Rohrmann S., Boeing H., Pischon T., Psaltopoulou T., Trichopoulou A., Trichopoulos D., Palli D., Vineis P., Tumino R., Berrino F., Kiemeney L.A.L.M., Bueno-De-Mesquita H.B., Quiros J.R., Gonzalez C.A., Martinez C., Larranaga N., Chirlaque M.D., Ardanaz E., Stattin P., Hallmans G., Khaw K.T., Bingham S., Slimani N., Ferrari P., Rinaldi S., Riboli E. (2007). Plasma carotenoids, retinol, and tocopherols and the risk of prostate cancer in the European Prospective Investigation into Cancer and Nutrition study. Am. J. Clin. Nutr..

[127] Peters U., Leitzmann M.F., Chatterjee N., Wang Y., Albanes D., Gelmann E.P., Friesen M.D., Riboli E., Hayes R.B. (2007). Serum lycopene, other carotenoids, and prostate cancer risk: a nested case-control study in the prostate, lung, colorectal, and ovarian cancer screening trial. Cancer Epidemiol. Biomark. Prev..

[128] Jian L., Lee A.H., Binns C.W. (2007). Tea and lycopene protect against prostate cancer. Asia-Pac. J. Clin. Nutr..

[129] Hwang E.-S., Lee H.J. (2006). Inhibitory effects of lycopene on the adhesion, invasion, and migration of SK-Hep1 human hepatoma cells. Exp. Biol. Med..

[130] Burgess L.C., Rice E., Fischer T., Seekins J.R., Burgess T.P., Sticka S.J., Klatt K. (2008). Lycopene has limited effect on cell proliferation in only two of seven human cell lines (both cancerous and noncancerous) in an in vitro system with doses across the physiological range. Toxicol. in Vitro.

[131] Müller K., Zucoloto S., Albuquerque Jr. R.F., Vannucchi H. (2007). Lack of inhibitory effect of lycopene on dysplastic lesions induced by 7,12-dimethyl-benz[a]anthracene in hamster buccal pouch. Nutr. Res..

[132] Huang J.P., Zhang M., Holman C.D.J., Xie X. (2007). Dietary carotenoids and risk of breast cancer in Chinese women. Asia-Pac. J. Clin. Nutr..

[133] Sesso H.D., Buring J.E., Zhang S.M., Norkus E.P., Gaziano J.M. (2005). Dietary and plasma lycopene and the risk of breast cancer. Cancer Epidemiol. Biomark. Prev..

[134] Larsson S.C., Bergkvist L., Näslund I., Rutegård J., Wolk A. (2007). Vitamin A, retinol, and carotenoids and the risk of gastric cancer: a prospective cohort study. Am. J. Clin. Nutr..

[135] Lian F., Smith D.E., Ernst H., Russell R.M., Wang X.-D. (2007). Apo-10′-lycopenoic acid inhibits lung cancer cell growth *in vitro*, and suppresses lung tumorigenesis in the A/J mouse model *in vivo*. Carcinogenesis.

[136] Guttenplan J.B., Chen M., Kosinska W., Thompson S., Zhao Z., Leonard A. (2001). Cohen Effects of a lycopene-rich diet on spontaneous and benzo[a]pyrene-induced mutagenesis in prostate, colon and lungs of the lacZ mouse. Cancer Lett..

[137] Coyne T., Ibiebele T.I., Baade P.D., Dobson A., McClintock C., Dunn S., Leonard D., Shaw J. (2005). Diabetes mellitus and serum carotenoids: Findings of a population-based study in Queensland, Australia. Am. J. Clin. Nutr..

[138] Polidori M.C., Mecocci P., Stahl W., Parente B., Cecchetti R., Cherubini A., Cao P., Sies H., Senin U. (2000). Plasma levels of lipophilic antioxidants in very old patients with Type 2 diabetes. Diabetes/Metabolism Res. Rev..

[139] Ylönen K., Alfthan G., Groop L., Saloranta C., Aro A., Virtanen S.M. (2003). Dietary intakes and plasma concentrations of carotenoids and tocopherols in relation to glucose metabolism in subjects at high risk of type 2 diabetes: The Botnia Dietary Study. Am. J. Clin. Nutr..

[140] Riso P., Brusamolino A., Martinetti A., Porrini M. (2006). Effect of a tomato drink intervention on insulin-like growth factor (IGF)-1 serum levels in healthy subjects. Nutr.Cancer.

[141] Wang L., Liu S., Pradhan A.D., Manson J.E., Buring J.E., Gaziano J.M., Sesso H.D. (2006). Plasma Lycopene, Other Carotenoids, and the Risk of Type 2 Diabetes in Wome. Am. J. Epidemiol..

[142] Kuhad A., Sethi R., Chopra K. (2008). Lycopene attenuates diabetes-associated cognitive decline in rats. Life Sci..

[143] Kumar P., Kumar A. (2009). Effect of lycopene and epigallocatechin-3-gallate against 3-nitropropionic acid induced cognitive dysfunction and glutathione depletion in rat: A novel nitric oxide mechanism. Food Chem. Toxicol..

[144] Akbaraly N.T., Faure H., Gourlet V., Favier A., Berr C. (2007). Plasma carotenoid levels and cognitive performance in an elderly population: Results of the EVA Study. J. Gerontol.-Ser. A.

[145] Gupta S.K., Trivedi D., Srivastava S., Joshi S., Halder N., Verma S.D. (2003). Lycopene attenuates oxidative stress induced experimental cataract development: An *in vitro* and *in vivo* study. Nutrition.

[146] Tang X.Y., Yang X.D., Wang L., Sun W.Q., Gut F., He H., Ding L., Qu S.L., Xiao C. (2006). To study the mechanisms and the effects of lycopene on lipid peroxidation injure in hyperlipemia rabbits. Atherosclerosis.

[147] Zhao Y., Yu W., Hu W., Yuan Y. (2003). Anti-inflammatory and anticoagulant activities of lycopene in mice. Nutr. Res..

[148] Saedisomeolia A., Wood L.G., Garg M.L., Gibson P.G., Wark P.A.B. (2009). Lycopene enrichment of cultured airway epithelial cells decreases the inflammation induced by rhinovirus infection and lipopolysaccharide. J. Nutr. Biochem..

[149] Lee C.-M., Chang J.-H., Moon D.-O., Choi Y.H., Choi I.-W., Park Y.-M., Kim G.-Y. (2008). Lycopene suppresses ovalbumin-induced airway inflammation in a murine model of asthma. Biochem. Biophys. Res. Commun..

[150] Sharma J.B., Kumar A., Kumar A., Malhotra M., Arora R., Prasad S., Batra S. (2003). Effect of lycopene on pre-eclampsia and intra-uterine growth retardation in primigravidas. Int. J. Gynecol. Obstet..

[151] Han C.H., Yang C.H., Sohn D.W., Kim S.W., Kang S.H., Cho Y.-H. (2008). Synergistic effect between lycopene and ciprofloxacin on a chronic bacterial prostatitis rat model. Int. J. Antimicrob. Agents.

[152] Forssberg A., Lingen C., Ernster L., Linberg O. (1959). Modification of the X-irradiation syndrome by lycopene. Exp. Cell Res..

[153] Riccioni G., Bucciarelli T., Mancini B., Di Ilio C., Della Vecchia R., D'Orazio N. (2007). Plasma lycopene and antioxidant vitamins in asthma: The PLAVA Study. J. Asthma.

[154] De Pablo P., Dietrich T., Karlson E.W. (2007). Antioxidants and other novel cardiovascular risk factors in subjects with rheumatoid arthritis in a large population sample. Arthritis Care Res..

[155] Trumbo P.R. (2005). Are there adverse effects of lycopene exposure?. J. Nutr..

[156] Christian M.S., Schulte S., Hellwig J. (2003). Developmental (embryo-fetal toxicity/teratogenicity) toxicity studies of synthetic crystalline lycopene in rats and rabbits. Food Chem. Toxicol..

[157] Shao A., Hathcock J.N. (2006). Risk assessment for the carotenoids lutein and lycopene. Regul. Toxicol. Pharm..

[158] Al-Wandawi H., Abdul-Rahman M., Al-Shaikhly K. (1985). Tomato processing wastes as essential raw materials source. J. Agric. Food Chem..

[159] Krishna G. (1985). Carotene and tocopherol in agro-industrial by-products and wastes of the tropics. Agric. Wastes.

[160] Benakmoum A., Abbeddou S., Ammouche A., Kefalas P., Gerasopoulos D. (2008). Valorisation of low quality edible oil with tomato peel waste. Food Chem..

[161] Naviglio D., Caruso T., Iannece P., Aragòn A., Santini A. (2008). Characterization of high purity lycopene from tomato wastes using a new pressurized extraction approach. J. Agric. Food Chem..

[162] Kassama L.S., Shi J., Mittal G.S. (2008). Optimization of supercritical fluid extraction of lycopene from tomato skin with central composite rotatable design model. Sep. Purif. Technol..

[163] Yang S.X., Shi W., Zeng J. (2004). Modelling the supercritical fluid extraction of lycopene from tomato paste waste using neuro-fuzzy approaches. Lect. Notes Comput. Sci..

[164] Xi J. (2006). Effect of high pressure processing on the extraction of lycopene in tomato paste waste. Chem. Eng. Technol..

[165] Jun X. (2006). Application of high hydrostatic pressure processing of food to extracting lycopene from tomato paste waste. High Pressure Res..

[166] Dhas P.H.A., John Zachariah T., Rajesh P.N., Subramannian S. (2004). Effect of blanching and drying on quality of mace (*Myristica Fragrans*). J. Food Sci. Technol..

[167] Choudhari S.M., Ananthanarayan L. (2007). Enzyme aided extraction of lycopene from tomato tissues. Food Chem..

[168] Sandei L., Leoni C. (2006). Exploitation of by-products (solid wastes) from tomato processing to obtain high value antioxidants. Acta Horticult..

[169] Lavecchia R., Zuorro A. (2008). Improved lycopene extraction from tomato peels using cell-wall degrading enzymes. Eur. Food Res. Technol..

[170] Nobre B.P., Pessoa F.L.P., Palavra A.F., Mendes R.L. Supercritical CO_2_ extraction of lycopene from tomato industrial waste. CHISA 2006-17th International Congress of Chemical and Process Engineering.

[171] Calvo M.M., García M.L., Selgas M.D. (2008). Dry fermented sausages enriched with lycopene from tomato peel. Meat Sci..

[172] Chiu Y.T., Chiu C.P., Chien J.T., Ho G.H., Yang J., Chen B.H. (2007). Encapsulation of lycopene extract from tomato pulp waste with gelatin and poly(γ-glutamic acid) as carrier. J. Agric. Food Chem..

[173] Baysal T., Ersus S., Starmans D.A.J. (2000). Supercritical CO2 extraction of β-carotene and lycopene from tomato paste waste. J. Agric. Food Chem..

[174] Altan A., McCarthy K.L., Maskan M. (2008). Evaluation of snack foods from barley-tomato pomace blends by extrusion processing. J. Food Eng..

[175] Schieber A., Stintzing F.C., Carle R. (2001). By-products of plant food processing as a source of functional compounds-recent developments. Trends Food Sci. Technol..

[176] Naviglio D., Pizzolongo F., Ferrara L., Naviglio B., Aragòn A., Santini A. (2008). Extraction of pure lycopene from industrial tomato waste in water using the extractor Naviglio. Afr. J. Food Sci..

[177] Knoblich M., Anderson B., Latshaw D. (2005). Analyses of tomato peel and seed byproducts and their use as a source of carotenoids. J. Sci. Food Agric..

[178] Lee M.T., Chen B.H. (2002). Stability of lycopene during heating and illumination in a model system. Food Chem..

[179] Chang C.H., Lin H.Y., Chang C.Y., Liu Y.C. (2006). Comparisons on the antioxidant properties of fresh, freeze-dried and hot-air-dried tomatoes. J. Food Eng..

[180] Kerkhofs N.S., Lister C.E., Savage G.P. (2005). Change in Colour and Antioxidant Content of Tomato Cultivars Following Forced-Air Drying. Plant Foods Human Nutr..

[181] Toor R.K., Savage G.P. (2006). Effect of semi-drying on the antioxidant components of tomatoes. Food Chem..

[182] Shi J., Le Maguer M., Kakuda Y., Liptay A., Niekamp F. (1999). Lycopene degradation and isomerization in tomato dehydration. Food Res. Int..

[183] Giovanelli G., Zanoni B., Lavelli V., Nani R. (2002). Water sorption, drying and antioxidant properties of dried tomato products. J. Food Eng..

[184] Hsu K.C. (2008). Evaluation of processing qualities of tomato juice induced by thermal and pressure processing. LWT-Food Sci. Technol..

[185] Goula A.M., Adamopoulos K.G., Chatzitakis P.C., Nikas V.A. (2006). Prediction of lycopene degradation during a drying process of tomato pulp. J. Food Eng..

